# Combining probability with qualitative degree-of-certainty metrics in assessment

**DOI:** 10.1007/s10584-018-2247-6

**Published:** 2018-07-26

**Authors:** Casey Helgeson, Richard Bradley, Brian Hill

**Affiliations:** 1 Earth and Environmental Systems Institute, Pennsylvania State University, 2217 Earth and Engineering Sciences Building, University Park, PA 16802 USA; 2 Department of Philosophy, Logic and Scientific Method and Centre for Philosophy of Natural and Social Science (CPNSS), London School of Economics, Lakatos Building Houghton Street, London, WC2A 2AE UK; 3 GREGHEC, CNRS, HEC Paris, 1 rue de la Libération, 78351 Jouy-en-Josas, France

## Abstract

Reports of the Intergovernmental Panel on Climate Change (IPCC) employ an evolving framework of calibrated language for assessing and communicating degrees of certainty in findings. A persistent challenge for this framework has been ambiguity in the relationship between multiple degree-of-certainty metrics. We aim to clarify the relationship between the *likelihood* and *confidence* metrics used in the Fifth Assessment Report (2013), with benefits for mathematical consistency among multiple findings and for usability in downstream modeling and decision analysis. We discuss how our proposal meshes with current and proposed practice in IPCC uncertainty assessment.

## Introduction

**Fig. 1:**
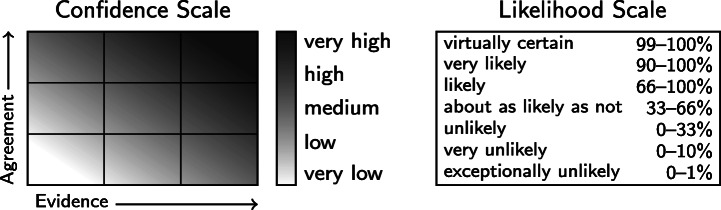
Confidence and likelihood scales for communicating degree of certainty in key findings of the IPCC AR5. Figures based on Mastrandrea et al. (2010)

Beginning with its Third Assessment Report (2001), characterization and communication of uncertainties by the Intergovernmental Panel on Climate Change (IPCC) has been informed by a series of *guidance notes* (Moss and Schneider 2000; Manning 2005; Mastrandrea et al. 2010) that share best practices and promote consistency across chapters and working groups. The guidance note for authors of the fifth and most recent assessment report (AR5) provides two “metrics for communicating the degree of certainty in key findings.” The first is *confidence*, a qualitative metric ranging from *very low* to *very high* and based on assessments of the underlying *evidence* (type, amount, quality, and consistency) and degree of *agreement* (Fig. 1, left). The second metric is probability, also called *likelihood*, and is conveyed through a menu of pre-defined terms, where, e.g., *unlikely* means 0–33% probability and *very unlikely* means 0–10% probability (Fig. 1, right).

Across assessment cycles and iterative refinements to the guidance notes, a persistent challenge for both authors and consumers of the reports has been confusion over *the relationship* between multiple degree-of-certainty scales (Kandlikar et al. 2005; Risbey and Kandlikar 2007; Swart et al. 2009; Shapiro et al. 2010; Jonassen and Pielke 2011; Mastrandrea and Mach 2011; Aven and Renn 2015; Mach et al. 2017).

In the AR5, this issue is most visible where authors use *both* confidence *and* likelihood terms together in a single statement, as seen, for example, in the findings on equilibrium climate sensitivity (ECS): “ECS is *likely* in the range 1.5 to 4.5 ^∘^C with *high confidence*. ECS is positive, *extremely unlikely* less than 1 ^∘^C (*high confidence*), and *very unlikely* greater than 6 ^∘^C (*medium confidence*)” (Stocker et al. 2013, 84). But the issue is really much wider, since even where confidence terms do not appear in the text, *all likelihood statements* should be read as confidence-qualified, where the implicit level of confidence is *high* or *very high* if otherwise unstated (Mastrandrea et al. 2010, criteria E, F). Deep down, all AR5 likelihood statements share the form of the ECS findings quoted above.

Post-AR5 commentary indicates continuing challenges for the interpretation and consistent usage of such findings (Aven and Renn 2015; Mach et al. 2017), with the most comprehensive analysis of uncertainty terms used across the AR5 concluding that “In practice and perhaps out of necessity, author teams adopted a spectrum of approaches in interpreting the relationship between confidence and likelihood. Redundancies and interactions across layers of characterized uncertainties were often handled differently and not transparently” (Mach et al. 2017, 9).

Heading into the sixth assessment cycle, we contribute a proposal to solve this particular challenge. We propose a simple mathematical model of the confidence–likelihood relationship that resolves outstanding ambiguities while respecting the qualitative nature of the confidence scale. Our proposal also preserves the conceptual distinction between the scientific estimate of chances (the likelihoods) and the evaluation of the evidential basis underpinning these estimates (the confidence assessments), which is an important motivation for reporting confidence as well as likelihood. In what follows, we briefly examine the interpretive problem a bit further, then present our proposal.

## Reasoning with likelihood and confidence

**Fig. 2:**
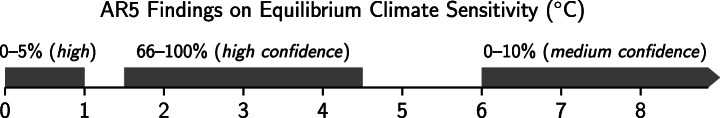
AR5 findings on equilibrium climate sensitivity (ECS). Probability ranges are the numerical translations of (from left to right) *extremely unlikely*, *likely*, and *very unlikely*

To further motivate the problem we mean to solve, we illustrate how ambiguity in the confidence–likelihood relationship can challenge attempts to reason systematically based on AR5 likelihood statements. Continuing with the ECS findings quoted above, note that the statements addressing the middle and left tail of the distribution are made with *high confidence* while the statement on the right tail is made with *medium confidence* (Fig. 2). A natural reading is that these confidence terms flag differences in the evidence base underpinning what can be said about one *value range* versus another: the evidence on small and middling values for ECS warrants *high confidence* whereas the evidence on more extreme values is weaker and allows for only *medium confidence*.

But this reading gets muddled when we try to draw out some of the mathematical consequences of the individual likelihood statements. For example, starting from the *medium confidence* statement on the right tail, assigning probability 0–.1 (*very unlikely*) to the range *ECS* > 6 is mathematically equivalent to assigning .9–1 (*very likely*) to *ECS* ≤ 6. Supposing that equivalent statements should enjoy the same level of confidence, we can equally regard the authors as reporting with *medium confidence* that ECS is *very likely* less than 6^∘^. But this new *medium confidence* statement covers the same low and midrange ECS values where the other findings say *high confidence* is appropriate. Are the original and derived statements consistent?

Or if we start from the *high confidence* finding on 1.5 < *ECS* < 4.5, the likelihood assignment .66–1 (*likely*) significantly constrains the probabilities of ECS values *outside* that range, as there is at most .33 probability left to go around. How much is leftover for *ECS* > 6, for example? Supposing that deductive consequences retain the confidence level of the statement from which they are derived, we can infer that ECS is (at most) *unlikely* (0–.33) greater than 6^∘^ (*high confidence*). But this contradicts our initial reading of the AR5 findings, according to which *medium* was the uniquely appropriate confidence term for statements addressing ECS values beyond 6.

If our initial reading is unpromising, then how should we interpret, and resolve the tension between, these overlapping and potentially competing *high* and *medium confidence* likelihood assignments?

## Proposal

We start from the idea that the underlying metrics *evidence* and *agreement* are best judged with respect to a concrete statement: evidence must be evidence *for something*, and agreement must be agreement *on something*. The head-scratching elicited above ultimately stems from an ambiguity in what that *something* is. Here, we explore the consequences of judging evidence and agreement, and hence confidence, with respect to a concrete *likelihood assignment* (such as *ECS* > 6 is *unlikely*).

One consequence of this move is that confidence now participates in a trade-off with *the precision* of the likelihood assignment: confidence can be raised (without going out and doing more research) by widening the probability interval; conversely, a likelihood assignment can be made sharper (more narrow) by accepting lower confidence. In terms of the evaluation of *evidence* and *agreement* that underwrites confidence assessments in the AR5 framework, wider, less informative probability intervals may enjoy greater confidence because they are supported by additional studies or lines of evidence from which sharper probabilistic conclusions cannot be drawn (*evidence*) and/or because broader statements generally make agreement between different lines of evidence easier (*agreement*).

And because confidence terms attach to the likelihood rather than the outcome directly, two findings can address the same outcome despite using different confidence levels. There is no *logical* inconsistency in reporting, for example, that the probability of ECS exceeding 6^∘^ is 0–.1 (*very unlikely*) with *medium confidence*, and 0–.33 (*unlikely*) with *high confidence*. The two statements *complement* one another, together giving an indication of the prevailing trade-off between confidence and precision. Informally, these findings say “We have good evidence that the probability is less than one tenth, and very strong evidence it is no more than than one third.” On this approach, there is no tension at all between the multiple findings discussed above (§2). All of those findings—both original and derived—can be understood as mutually consistent and complementary.

Usage in the AR5 conforms to this understanding of the confidence–likelihood relationship insofar as authors have sometimes traded off precision in the likelihood assignment against the level of confidence—a practice noted by Mach et al. (2017), calling it *adjustment of likelihood as a function of confidence*. We take our proposal to offer a clear rationale for such “adjustments,” and a route to making them more principled and more transparent.

One benefit of clarifying the confidence–likelihood relationship is that it now becomes possible to define what constitutes *logical consistency* among multiple findings. What is needed to define consistency is an overarching mathematical structure that can relate one finding to another and systematize the logical constraints that each finding puts on the others.

When working with *exact probabilities*, the numbers assigned to different outcome ranges ought to be consistent with a single probability density function (pdf). But here, we want to address *probability intervals*. The analogous construct for probability intervals is a *set* of pdfs. Assigning probability 0–.1 to outcome *x* means that within the set of pdfs collectively representing authors’ uncertainty, the smallest probability given to outcome *x* by any pdf is 0 and the largest probability given to *x* by any pdf is .1. (Equivalently, we can say that 0 and .1 are the narrowest bounds such that every pdf in the set agrees on the probability of *x* falling within the interval.)

**Fig. 3:**
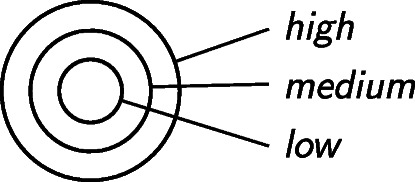
Each confidence level is associated with its own set of probability distributions. The nested structure reflects the trade-off between confidence and the precision of likelihood assignments

So a set of pdfs systematizes, and enforces consistency among, the probability intervals assigned to different ranges of a single quantity such as ECS. The question is how *confidence levels* fit into the picture. We associate each level of confidence *with its own set of pdfs*, where higher-confidence sets encompass lower-confidence sets (Fig. 3). This nesting of sets naturally encodes the trade-off between confidence and precision, since more inclusive sets of pdfs translate to wider probability intervals for any given outcome. Multiple likelihood-plus-confidence findings addressing the same uncertain quantity are mutually consistent if and only if they can be modelled by such a mathematical structure.

This *conceptual* clarification of what constitutes logical consistency provides for the option of an in-practice *consistency check*, where such a quality control mechanism (Parker and Risbey 2015) is desirable and practical. (This would typically require consideration of at most two nested sets.) A clear standard of consistency is, moreover, a prerequisite for employing more formal expert elicitation protocols, as recommended by the TAR, AR4, and AR5 guidance notes as well as a chorus of commentators (Reilly et al. 2001; Oppenheimer et al. 2007; Shapiro et al. 2010; Moss 2011; Yohe and Oppenheimer 2011; Morgan 2014; Thompson et al. 2016; Oppenheimer et al. 2016; Mach et al. 2017).

Our proposal also helps answer the worry that qualitative metrics like confidence are unhelpful because whatever information they convey cannot be systematically propagated to “downstream” modeling and decision analysis (e.g., Morgan 2014). By building confidence assessments into a formal belief representation (the nested sets), we facilitate this propagation. The decision theory literature has developed and defended a range of decision models that reserve a principled role for something like confidence assessments (Gilboa and Marinacci 2013; Hill 2013; Bradley 2017); our proposal enables a bridge from IPCC uncertainty assessment to this literature—the most direct link (see Bradley et al. 2017) pointing to a model of confidence-based decision from Hill (2013, forthcoming). Similar constructs have also been defended in econometrics (Manski and Nagin 1998; Manski 2013) and decision analysis (Ben-Haim 2006).

Note that while we have so far spoken in terms of continuous underlying probability distributions (pdfs), all of the above applies equally where such precision is unrealistic or unhelpful. In particular, nothing we have said should be understood to constrain authors’ prerogative to choose the appropriate degree of specificity and precision when describing possible events or outcomes. Consider, for example, the finding that “Monsoon onset dates are *likely* to be early or not to change much” (Christensen et al. 2013, 1229). This “likely” can be understood in terms of a set of discrete probability distributions on the simple two-way partition of monsoon onset dates: *early or little change* versus *substantial delay*. Probability .66–1 for the former means 0–.33 for the latter. These likelihood assignments implicitly carry *high* or *very high* confidence, and they are potentially consistent with additional, complementary lower-confidence statements giving sharper likelihood estimates.

While warnings against assigning probability to ambiguously defined events still apply (e.g., Shapiro et al. 2010; Budescu et al. 2012), neither broad outcome characterizations (the simple two-way partition), fuzzy boundaries (“early or little change”), nor imprecisely defined phenomena (“monsoon”) formally undermine the applicability of the conceptual machinery our proposal offers. These and other features may, however, influence the degree to which *engagement with that machinery* by IPCC authors is worthwhile in any given instance—which will, naturally, vary across topics and findings.

## Discussion

To provide context and highlight issues for further discussion, we note points of contrast between our proposal and two existing recommendations for IPCC practice: (1) the *sequential assessment* of confidence followed by likelihood that is implied in the AR5 guidance (Mastrandrea et al. 2010) and explicit in Mastrandrea et al. (2011), and (2) a recommendation from Mach et al. (2017)—looking forward to the AR6—to foreground *quantitative* characterization of uncertainties by making presentation of *only the likelihood assignment* the preferred reporting option for IPCC findings. We discuss these in turn.

Regarding the sequential procedure (1), systematically assessing confidence *before likelihood enters the picture* makes that assessment a function of the outcome range alone. It follows that a given outcome range (like *ECS* > 6) must be associated with a *fixed confidence level*, which in turn rules out trade-offs between confidence and precision of likelihood. But it is precisely through legalizing such trade-offs that our proposal allows multiple findings to coherently address the same outcome using different confidence terms, thus resolving the apparent contradictions that arise when trying to reason from AR5-style findings (the ECS example, §2). Such “same-outcome” findings cannot be reconciled within the constraints imposed by a strictly sequential, confidence-first procedure. Competition between stated and derived findings must instead be averted by restricting either what can be stated or what can be derived. Both risk relinquishing any meaningful characterization of the evidence and agreement underpinning a likelihood assignment.

To prevent contradictions through restricting what can be stated, one could prohibit the use of different confidence terms for different ranges of the same uncertain quantity. Were medium confidence used on the mode of ECS as well as the tail, then likelihood constraints derived from the mode and applied to the tail would carry medium confidence (not high confidence as in our §2 derivation)—in line with the confidence level already assigned to the tail. But this solution imposes an artificial constraint that restricts otherwise appropriate and helpful use of the qualitative metric. It also raises questions about the meaning and validity of “evidence” and “agreement” when those assessments are forced to remain constant across outcomes ranges and likelihood judgements.

To prevent contradictions by restricting what can be derived, one must deny that a likelihood assignment and its consequences enjoy the same level of confidence. If probability constraints derived from assigned likelihoods have indeterminate confidence, then our §2 illustration fails to generate a confidence-level conflict on the tail of the distribution. So you can preserve the option of different confidence terms for different outcome ranges only at the cost of making those terms (so to speak) *non-transferrable* to the mathematical consequences of a likelihood assignment. This solution gives up altogether on propagating qualitative assessments into further reasoning, modeling and decision analysis.

This brings us to *likelihood only* as the preferred reporting option (2). One rationale for (2) might be the thought that likelihood—when used—displaces and supplants confidence such that the qualitative language contains no additional information beyond what is now expressed by the likelihood. This rationale conflicts with our proposal. When used in conjunction with likelihood, we understand confidence to express something like Keynes’ (1921/1973) “weight of evidence” behind a probability statement, where the weight he refers to includes the quantity, quality and diversity of evidence underpinning a claim. A key feature of this notion is that the same probability statement can convey a different overall picture of uncertainty when underpinned by different “weights.” For example, the *likely* range for ECS given by the AR5 (1.5 to 4.5 ^∘^C) is the same as that given by the First (1990), Second (1995), and Third (2001) Assessment Reports (Cubasch et al. 2001, 67). But the body of research underpinning those likelihood statements grew between 1990 and 2013. This difference can be expressed through confidence assessments.

Confidence assessments not only add information about the state of scientific understanding in some domain, they may also be relevant for policy making that draws on this science. Consider two potential policies, each of which is justified by scientific findings reported with the same likelihood level, but underpinned by different amounts of evidence and agreement, and hence enjoying differing degrees of confidence. It seems reasonable in such cases to prioritize the policy backed up by evidence-rich, high-confidence findings. Likelihood-only reporting cannot support such a practice.

Yet even if confidence terms do contain substantive and decision-relevant information beyond what is conveyed by the likelihood, one might still worry that this information is too difficult for consumers of the reports to interpret and make use of. One response to these worries—and the one embodied by our proposal—is to clarify the structure of the qualitative assessments such that the information they are meant to convey might be transmitted more cleanly and used more systematically. Indeed, our proposal connects neatly into decision-making approaches that incorporate confidence into the evaluation of options (Hill forthcoming; Bradley 2017). (Of course, this theoretical point about the possibility and relevance of consistent confidence reporting does not preclude *de-emphasizing* confidence terms in the text of the report—perhaps pushing them into the traceable account—where the benefits of simplicity and readability outweigh the cost of losing the confidence information.)

## Conclusion

A persistent shortcoming of the IPCC’s evolving expert judgement framework has been the ambiguous relationship between multiple uncertainty scales. The issue arises in the AR5 between the confidence and likelihood scales, contributing to difficulties both in applying the framework and interpreting the findings. We have proposed a rigorous, principled solution that resolves outstanding ambiguities while also enabling mathematical reasoning from individual likelihood assignments and providing a consistency check on sets of likelihood assignments.

Designing an uncertainty framework for use in assessment involves striking a balance between a host of important considerations, many of which have not been addressed here (see, e.g., Yohe and Oppenheimer 2011; Adler and Hadorn 2014; Budescu et al. 2014; Mach and Field 2017). More pliable language eases application across diverse fields of study and provides wiggle room for resolving expert disagreements under the formidable time and logistical pressures faced by IPCC authors. And improvements in terminological rigor that add to authors’ procedural obligations may impose opportunity costs elsewhere, for example cutting into the breadth or depth of literature authors are able to assess. While it is important to acknowledge these many trade-offs, we have not attempted to navigate them here. Our aim was simply to clarify one consideration—the conceptual relationship between confidence and likelihood—under the conviction that such clarifications can only improve reflection on current and future practice. We offer our proposal with the aims of stimulating further discussion and highlighting the importance of maintaining logical consistency among findings, communicating the weight of evidential support, and enabling downstream use of assessment outputs.
